# The animacy advantage in memory occurs under self-paced study conditions, but participants’ metacognitive beliefs can deter it

**DOI:** 10.3389/fpsyg.2023.1164038

**Published:** 2023-05-11

**Authors:** Michael J. Serra, Carlee M. DeYoung

**Affiliations:** Department of Psychological Sciences, Texas Tech University, Lubbock, TX, United States

**Keywords:** animacy advantage, animacy effect, adaptive memory, free recall (memory), self-paced study, metacognitive beliefs

## Abstract

**Introduction:**

Animacy distinguishes living (animate) things from non-living (inanimate) things. People tend to devote attention and processing to living over nonliving things, resulting in a privileged status for animate concepts in human cognition. For example, people tend to remember more animate than inanimate items, a phenomenon known as the “animacy effect” or “animacy advantage.” To date, however, the exact cause(s) of this effect is unknown.

**Methods:**

We examined the animacy advantage in free-recall performance under computer-paced versus self-paced study conditions and using three different sets of animate and inanimate stimuli (Experiments 1 and 2). We also measured participants’ metacognitive beliefs (expectations) about the task before it began (Experiment 2).

**Results:**

We consistently obtained an animacy advantage in free-recall, regardless of whether participants studied the materials under computer-paced or self-paced conditions. Those in self-paced conditions spent less time studying items than did those in computer-paced conditions, but overall levels of recall and the occurrence of the animacy advantage were equivalent by study method. Importantly, participants devoted equivalent study time to animate and inanimate items in self-paced conditions, so the animacy advantage in those conditions cannot be attributed to study time differences. In Experiment 2, participants who believed that inanimate items were more memorable instead showed equivalent recall and study time for animate and inanimate items, suggesting that they engaged in equivalent processing of animate and inanimate items. All three sets of materials reliably produced an animacy advantage, but the effect was consistently larger for one set than the other two, indicating some contribution of item-level properties to the effect.

**Discussion:**

Overall, the results suggest that participants do not purposely allocate greater processing to animate over inanimate items, even when study is self-paced. Rather, animate items seem to naturally trigger greater richness of encoding than do inanimate items and are then better remembered, although under some conditions participants might engage in deeper processing of inanimate items which can reduce or eliminate the animacy advantage. We suggest that researchers might conceptualize mechanisms for the effect as either centering on intrinsic, item-level properties of the items or centering on extrinsic, processing-based differences between animate and inanimate items.

## 1. Introduction

Animacy refers to the qualities that distinguish animate (living) things from inanimate (non-living) things, or the qualities that make something seem alive (VanArsdall and Blunt, 2022). Examples of living things are animals (hedgehog) and humans (dancer), while nonliving things include natural objects (rock) and man-made objects (plate). People tend to preferentially devote attention and processing to living over nonliving things in their environment and in their thoughts, and this tendency in turn affects many other aspects of human cognition, including attention (New et al., 2007; Altman et al., 2016; Bugaiska et al., 2019), perception (Scholl and Tremoulet, 2000), language (Vihman and Nelson, 2019), numerical processing (Zanini et al., 2020), metacognitive monitoring (Li et al., 2016; DeYoung and Serra, 2021), and memory (Popp and Serra, 2016; Nairne et al., 2017). Most relevant, in many memory tasks, people tend to remember more animate than inanimate items, a phenomenon known as *the animacy effect* or *the animacy advantage*. In the present experiments, we compared the occurrence of the animacy advantage for free-recall performance under computer-paced versus self-paced study conditions and using three different sets of stimuli.

The animacy advantage in memory can occur in recognition tasks (e.g., VanArsdall et al., 2013; Bonin et al., 2014; Leding, 2020) and cued-recall tasks (e.g., VanArsdall et al., 2015; Popp and Serra, 2016; DeYoung and Serra, 2021; but see Popp and Serra, 2016; Kazanas et al., 2020; Serra and DeYoung, 2023), but to date researchers have most often examined the effect in the context of free-recall (*cf.* Nairne et al., 2013, 2017; Bonin et al., 2015; Li et al., 2016; Popp and Serra, 2016, 2018; Gelin et al., 2017, 2019; VanArsdall et al., 2017; Leding, 2018, 2019; Meinhardt et al., 2018, 2020; Félix et al., 2019; Serra, 2021). In free-recall tasks, participants usually study a list of words, one at a time, and then try to recall the words from memory without hints or assistance. Different researchers have found the effect with different sets of words, different numbers of words per list, and different numbers of study trials. It occurs with both pure lists (only animate words versus only inanimate words) and mixed lists (both animate and inanimate words) of to-be-remembered words (Popp and Serra, 2016), with and without the inclusion of buffer words or a distractor task (e.g., Nairne et al., 2013; Popp and Serra, 2016), and across the serial-positions of the list (Serra, 2021).

The discovery of and initial accounts of the animacy advantage in memory stemmed from concepts in evolutionary psychology (*cf.* Nairne et al., 2013, 2017; VanArsdall et al., 2013, 2015). Specifically, the tendency of our attention and memory systems to prioritize animate over inanimate things in our environment might have stemmed from fitness pressures faced by our early ancestors to quickly detect threats in the environment (predators, competitors) or to remember where sources of food or shelter were located. These tendencies might still exist today because they are deeply ingrained in our evolutionary heritage. Such ideas are quite viable: it is apparent that physical memory mechanisms can be conserved through evolution (Alberini, 1999), and different mouse strains exhibit differences in learning and memory performance (Wehner and Silva, 1996; Crawley et al., 1997). Genetic analyses are important and support adaptive accounts, but it is difficult to directly test the assumptions of such “ultimate” accounts using behavioral methods. Instead, behavioral researchers focus on testing “proximate” mechanisms for the effects of animacy on memory, testing mechanisms that can produce the effects now in modern humans, regardless of the ultimate origins of such tendencies.

Researchers have examined several potential proximate mechanisms that could cause or contribute to the animacy advantage in memory, but the exact cause(s) of the effect in free-recall performance is not yet apparent. The effect *does not* seem to occur because, compared to inanimate words, animate words are more threatening (Leding, 2019, 2020), more arousing (Meinhardt et al., 2018; Popp and Serra, 2018; Leding, 2019), more easily categorizable (Gelin et al., 2017; VanArsdall et al., 2017; Serra, 2021), or more likely to invoke mental imagery (Gelin et al., 2019; Blunt and VanArsdall, 2021; but see Bonin et al., 2015). Despite ample evidence that animate items attract attention compared to inanimate items (e.g., Altman et al., 2016; Bugaiska et al., 2019), research has not consistently found a relationship between attention capture and the animacy advantage in free-recall performance (*cf.* Bonin et al., 2015; Leding, 2019; Rawlinson and Kelley, 2021). It does not seem that most participants know about the animacy advantage (DeYoung and Serra, 2021; but see Li et al., 2016) or actively cause it to occur by purposely allocating greater processing to animate over inanimate items (Serra, 2021), but they *can* alter how they process animate and inanimate items to cause the effect to be larger or smaller if the task instructions lead them to do so (*cf.* DeYoung and Serra, 2021; Shull et al., n.d.). Some currently viable accounts of the animacy advantage in memory suggest that the effect could occur because animate items naturally trigger greater richness of encoding than do inanimate items, perhaps because animate items activate more related information (Meinhardt et al., 2020; Bonin et al., 2022) or have more semantic features (Rawlinson and Kelley, 2021). Of course, more than one factor could simultaneously contribute to the effect (Rawlinson and Kelley, 2021; Leding, 2022). For example, Meinhardt et al. (2020) suggested that animate items might first capture attention compared to inanimate items, which then leads to the preferential and deeper processing of animate items over inanimate items, leading to the memory difference.

The purpose of the present experiments was to examine the animacy advantage in free-recall performance using some novel methodological conditions for this topic. Most prior demonstrations of the animacy advantage in free-recall performance have involved the experimenter-paced (i.e., computer-paced) presentation of the stimuli during encoding. In both Experiments 1 and 2, we compared the effect under computer-paced and self-paced study conditions (between-participants). Given that processing-based differences between animate and inanimate items seem to contribute to the occurrence of the animacy advantage under computer-paced study conditions (i.e., Meinhardt et al., 2020; Rawlinson and Kelley, 2021; Bonin et al., 2022), self-paced study conditions could allow a greater opportunity to observe such differences. In Experiment 2, we measured participants’ metacognitive beliefs about the animacy advantage just before they began the task to allow for a more fine-grained consideration of how people study the items.

Most often, participants devote greater study time to more difficult items than easier items (e.g., Nelson and Leonesio, 1988; Thiede and Dunlosky, 1999; Metcalfe, 2002). Such effects can be enhanced after participants gain experience with the items or task. For example, participants who gained experience with the effects of serial position on free-recall performance under computer-paced conditions later devoted greater study time to items in the middle of the list (those that were least likely to be learned) under self-paced study conditions (Murphy et al., 2022). Therefore, one possibility is that participants might devote more study time to inanimate over animate items, leading to a reduced memory advantage for animate over inanimate items, or perhaps even no difference or an *inanimate* memory advantage (*cf.* DeYoung and Serra, 2021; Shull et al., n.d.). A related possibility is that, as participants gain experience with the animacy advantage, they might begin to devote greater study time to inanimate than animate items across study-test trials. In Experiment 2, participants might devote more study time to the type of item they believe is *more difficult* to remember (i.e., to counteract expected differences by animacy), regardless of whether that belief reflects an animate or inanimate advantage.

In contrast, in some situations, participants devote more study time to items they perceive as easier to learn (Metcalfe, 2002; Serra and Dunlosky, 2010; Magreehan et al., 2016) or that have greater actual or perceived value (Dunlosky and Thiede, 1998; Murphy et al., 2023). As such, another possibility is that participants might devote more study time to animate over inanimate items, leading to a large memory advantage for animate over inanimate items (perhaps even larger than occurs under computer-paced conditions). In Experiment 2, specifically, participants might devote more study time to the type of item they believe is *easier* to remember, potentially causing the animacy advantage to become larger than normal if they believe that animate items are more memorable than inanimate items (*cf.* Shull et al., n.d.). In contrast, participants who believe that inanimate items are more memorable than animate items might devote study time preferentially to inanimate over animate items, reducing or even reversing the typical animacy advantage.

Of course, a third possibility is that participants in the self-paced conditions will devote study time equally to animate and inanimate items and the animacy advantage will occur anyway as in computer-paced conditions, which would be consistent with prior evidence that participants do not seem to purposely produce the animacy advantage by devoting greater or differential processing to animate over inanimate items (DeYoung and Serra, 2021; Serra, 2021). Such an outcome would also support the idea that item-level or processing-based differences between animate and inanimate items contribute to the animacy advantage, but likely in an unconscious way (DeYoung and Serra, 2021; Rawlinson and Kelley, 2021; Serra, 2021).

Finally, we also used three different sets of materials from earlier studies (i.e., Nairne et al., 2013; Popp and Serra, 2016, 2018) in the present experiments. By directly comparing the size of the animacy advantage for different sets of materials within the same experiments and samples, we can consider whether item-level properties of animate and inanimate items contribute to the effect in a bottom-up way, likely outside of participants’ awareness or control (*cf.* DeYoung and Serra, 2021; see Serra and DeYoung, 2023, for a similar examination using paired-associates materials). To our knowledge, these are the first studies to directly compare the size of the animacy advantage in free-recall performance for different sets of materials within the same sample or experiment.

## 2. Experiment 1

### 2.1. Materials and method

#### 2.1.1. Participants

The participants were 210 undergraduate college students from the psychology participant pool at Texas Tech University. They participated for class credit.

We used PANGEA (“Power ANalysis for GEneral Anova designs”) ver. 0.2 (Westfall, 2016) to perform power analysis for the present design. Several prior studies have demonstrated effects of animacy on free-recall performance yielding an effect size of *Cohen’s d* = 0.4 or greater when using a single study-test trial of free-recall. Our studies, however, examined this effect over three study-test trials of the same items [as in Nairne et al. (2013)], which adds statistical power. Using just this aspect of the design (animacy by trial) and assuming an effect size of *Cohen’s d* = 0.4 or greater for the main effect of animacy on recall, we would need 35 participants per group to have sufficient power (above 0.8) to detect such an effect within any single group. Therefore, this is the per-group sample size we used in both experiments (35 participants per group multiplied by six groups = 210 participants). That said, the present experiments also included two between-participants variables of interest: study method and list source. With 35 participants in each of the six groups, we would be sufficiently powered (above 0.8) to detect an overall effect size of animacy or trial on memory of *Cohen’s d* = 0.17 or higher. More important, we would be sufficiently powered (above 0.8) to detect an effect size of list on memory of *Cohen’s d* = 0.25 or higher, and an effect size of study method on memory of *Cohen’s d* = 0.20 or higher.

We neglected to obtain demographics data for the participants in the present two experiments. That said, our participant pool is typically about 70% female and 30% male, of a mean age around 19 years old, and approximately 70% white or Caucasian, 20% Hispanic or Latin, and the rest identifying as another race or ethnic group (or as more than one race or ethnic group). Our samples likely had similar demographics.

#### 2.1.2. Materials

The study materials were three lists of animate words (e.g., duck, soldier, turtle) and inanimate words (e.g., hat, rake, violin) from three previously published papers on this topic: a list of 12 animate and 12 inanimate words from Nairne et al. (2013), a list of 84 animate and 84 inanimate words from Popp and Serra (2016), and a list of 40 animate and 40 inanimate words from Popp and Serra (2018). Some words appeared on more than one of the lists, but we assigned each participant to only study words from one list, so no word ever appeared more than once per participant. The word lists all appear in their entirety in the original papers, including values for the factors on which those authors balanced the animate and inanimate word lists. For ease of access and direct comparison, however, we provide summaries of these lists’ properties in Table 1.

**Table 1:** Attributes of the word lists.

	Animate words		Inanimate words	
	M	SD	M	SD
Nairne et al. (2013) List				
Age of Acquisition	2.8	1.0	2.7	0.8
Category Size	22.3	5.9	23.2	6.0
Category Typicality	0.2	0.2	0.2	0.2
Concreteness	593	29	592	17
Familiarity	504	70	507	31
Imagery	589	37	578	30
Kučera-Francis Freq.	21.7	23	16.5	16
Meaningfulness	448	56	438	32
Number of Letters	5.3	1.8	5.0	1.4
Relatedness	0.1	0.1	0.1	0.1
Popp and Serra (2016) List				
Concreteness	6.3	0.3	6.3	0.2
Google Frequency	126.8 × 10^6^	200.8 × 10^6^	135.2 × 10^6^	118.8 × 10^6^
Imagery	6.1	0.5	6.1	0.3
Number of Letters	6.1	1.9	6.3	1.7
Popp and Serra (2018) List				
Age of Acquisition	7.0	2.4	7.4	2.7
Arousal	4.0	0.7	4.0	0.8
Concreteness	4.6	0.3	4.6	0.2
Dominance	5.4	0.5	5.4	0.6
Google Frequency	187.8 × 10^6^	324.7 × 10^6^	215.6 × 10^6^	267.4 × 10^6^
Number of Letters	6.7	1.6	6.6	1.4
Valence	5.6	0.9	5.3	0.9

We also identified four words that were not on any of the word lists to serve as primacy and recency buffers. Specifically, for all participants, the words “goose” and “fork” were always the first two words presented on every trial, and the words “spoon” and “deer” were always the last two words presented on every trial, but we did not count participants’ recall of these words (*cf.* Nairne et al., 2013). Prior research indicates that the animacy advantage in free recall is persistent across serial position (Serra, 2021), so excluding these words from analysis is not likely to alter the occurrence of the effect over the rest of the list.

The materials also included a custom computer program that presented all items for study and recorded participants’ free-recall performance, as well as performed the consent process, and provided the instructions at the start of the task and a debriefing at the end. We created this program using LiveCode Ltd. (2019).

#### 2.1.3. Design

The study involved a 2 (animacy: animate vs. inanimate words) × 3 (trial: Trial 1 vs. Trial 2 vs. Trial 3) × 3 (word list source: Nairne et al., 2013 vs. Popp and Serra, 2016 vs. Popp and Serra, 2018) × 2 (study method: computer-paced vs. self-paced) mixed design. Word list and study method were between-participants factors; animacy and trial were within-participants factors.

#### 2.1.4. Procedure

We based the procedure closely on that of Nairne et al. (2013), Study 2, see also the experiments in VanArsdall et al. (2017). For the conditions that studied the words from Nairne et al. (2013) with computer-paced study, the procedure was therefore nearly identical to the procedure in that original study except for the choice of the specific primacy and recency buffer items (the recall of which we did not score, as in that study).

Prior to the start of the session, we randomly assigned each participant to study words from one of the three lists and either under computer-paced or self-paced study conditions, with the restriction that we eventually assigned the same number of participants (35) to each of the six groups. When the procedure started, the computer program randomly chose 10 animate words and 10 inanimate words from the designated word list to serve as the participant’s items for all three study trials. Participants in the same list condition were therefore unlikely to study the same exact subset of words as each other, although obviously there was less room for variance with the shorter Nairne et al. (2013) list than with the longer lists from Popp and Serra (2016) and Popp and Serra (2018). The program then conducted the informed-consent process and provided written instructions to the participants. The instructions explained that participants would study two-dozen items for a free-recall test, over three study-test trials, but made no mention of the animacy of the words. The instructions noted that participants did not need to recall the words in the order studied. The instructions informed participants in the computed-paced conditions that each word would appear on screen “for a few seconds,” but informed participants in the self-paced conditions that they would control the study time of each item by clicking an on-screen icon to proceed to the next item.

The program then began the first study phase. Regardless of word list condition, the program first presented the two primacy buffer words, with a 250 millisecond interitem interval. The program then presented the participants’ actual 20-word list, one at a time in a fully randomized order, with a 250 millisecond interitem interval. Finally, the program presented the two recency buffer words, one at a time, with a 250 millisecond interitem interval. For participants in the computer-paced conditions, buffer words and target words appeared on screen for study for five seconds each. For participants in the self-paced conditions, participants controlled how long a buffer or target word appeared on screen for study by clicking an on-screen icon to proceed to the next item. The computer program recorded the self-paced study time for each non-buffer item.

After studying all the words, participants completed a 60 s distracter task as in Nairne et al. (2013): the program showed the participant a random whole-number digit from 1 to 8 and the participant clicked an on-screen button to indicate whether the number shown was an odd or even number. After the distracter task, participants attempted to recall the words they previously studied by typing them into a field on the computer screen. The computer program displayed words already entered to the participants but did not provide any feedback regarding correctness. When participants felt they could not recall any more words, they clicked an icon on the screen to continue.

The procedure then repeated for two more trials, using a new random ordering for the 20 critical words on the subsequent study phases (but maintaining the same primacy and recency buffer items). After completing the third test, participants read a debriefing on the computer screen and then the researcher dismissed them.

### 2.2. Results

The data for Experiment 1 is available at https://osf.io/6kndh/.

#### 2.2.1. Self-paced study time

**Figure 1:**
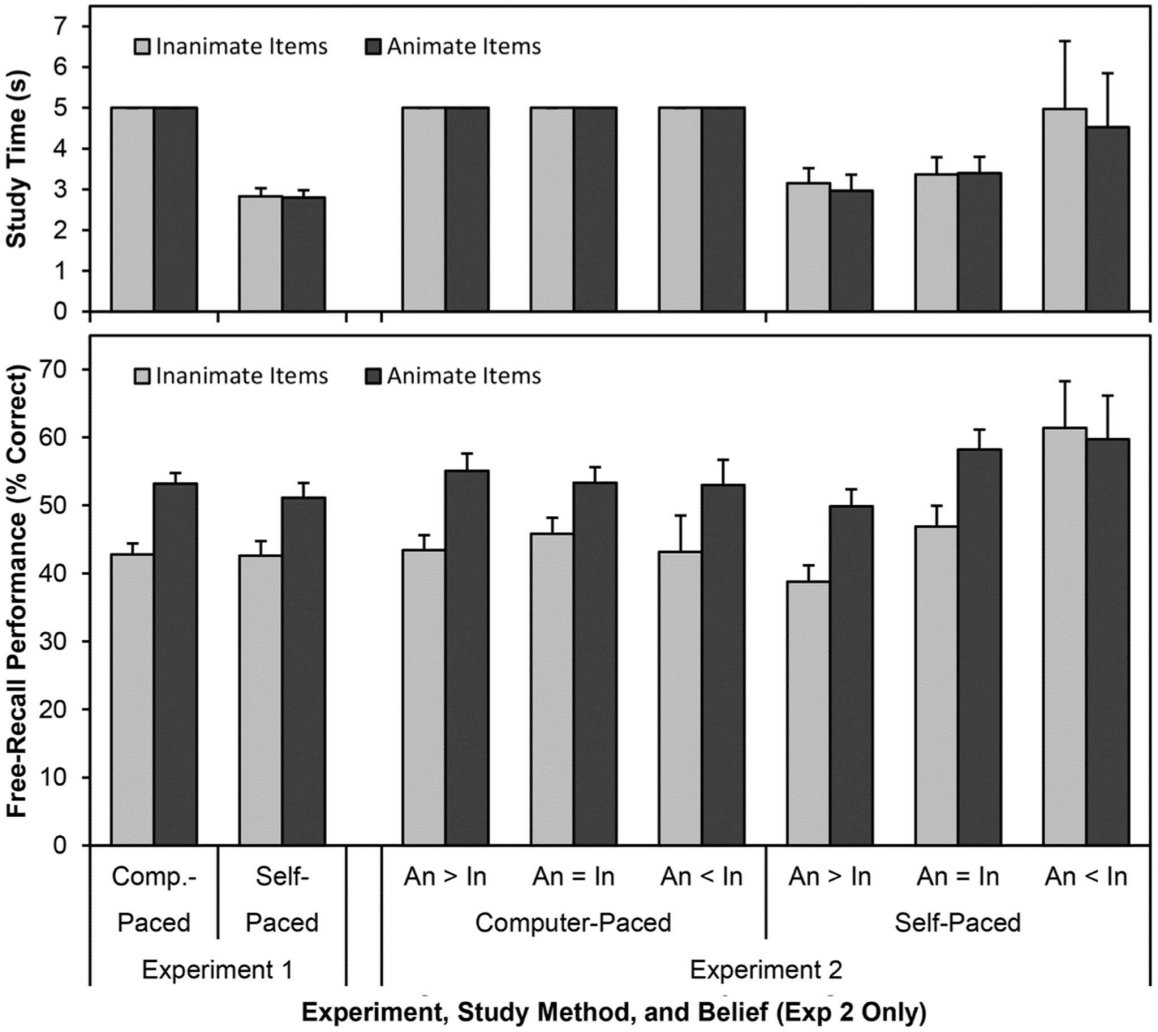
The mean study time (in seconds, top panel) and free-recall performance (percent recalled, bottom panel) for animate and inanimate words in Experiments 1 and 2, split by study method (computer-paced and self-paced). Results are collapsed on trial and list source. The results for Experiment 2 are split based on participants’ self-reported beliefs about the effects of animacy on memory: animate items are more memorable than inanimate items (“An > In”), animate items are equally as memorable as inanimate items (“An = In”), and animate items are less memorable than inanimate items (“An < In”). Error bars are one standard error of the mean.

We calculated the mean study time (measured in milliseconds then converted to seconds) for those participants in the self-paced conditions for animate and inanimate items on the three study trials (Table 2; Figure 1). We analyzed study time with a 2 (animacy: animate vs. inanimate words) × 3 (trial: Trial 1 vs. Trial 2 vs. Trial 3) × 3 (word list source: Nairne et al., 2013 vs. Popp and Serra, 2016 vs. Popp and Serra, 2018) mixed ANOVA. Self-paced study time did not differ by animacy, *F*(1,102) = 0.103, *MSE* = 1.645, *p* = 0.749, *η_p_^2^* < 0.01, or by list, *F*(2,102) = 0.207, *MSE* = 21.382, *p* = 0.813, *η_p_^2^* < 0.01. Polynomial contrasts indicated that self-paced study time decreased both linearly, *F*(1,102) = 80.322, *MSE* = 18.060, *p* < 0.001, *η_p_^2^* = 0.44, and quadratically, *F*(1,102) = 37.149, *MSE* = 6.095, *p* < 0.001, *η_p_^2^* = 0.27, across the study trials. None of the interactions were significant.

**Table 2:** Mean study time and mean free-recall performance in experiment 1.

List and trial	Computer-paced conditions				Self-paced conditions			
	Inanimate		Animate		Inanimate		Animate		M	SD	M	SD	M	SD	M	SD
Study time (in seconds)
Nairne et al. (2013) List								
Trial 1	5	–	5	–	5.23	5.04	5.54	4.41
Trial 2	5	–	5	–	2.07	2.14	1.80	1.30
Trial 3	5	–	5	–	1.71	1.22	1.51	1.05
Popp and Serra (2016) List								
Trial 1	5	–	5	–	5.07	4.38	5.47	5.33
Trial 2	5	–	5	–	1.86	1.44	1.87	1.73
Trial 3	5	–	5	–	1.11	0.68	1.23	0.91
Popp and Serra (2018) List								
Trial 1	5	–	5	–	4.77	5.05	4.51	3.77
Trial 2	5	–	5	–	2.24	1.68	1.96	1.13
Trial 3	5	–	5	–	1.42	1.09	1.30	0.69
Free-recall performance (% correct)
Nairne et al. (2013) List								
Trial 1	24.05	17.36	38.10	15.56	27.38	21.54	39.05	23.46
Trial 2	43.81	20.74	58.57	20.76	40.71	24.73	57.86	21.57
Trial 3	56.19	20.45	68.76	21.96	52.86	23.65	64.05	23.20
Popp and Serra (2016) List								
Trial 1	24.76	17.33	36.43	16.05	35.71	27.31	42.14	27.19
Trial 2	45.48	20.14	54.29	20.15	46.90	28.66	53.81	27.74
Trial 3	56.90	24.63	64.05	20.69	56.43	29.43	59.29	29.83
Popp and Serra (2018) List								
Trial 1	25.00	9.26	32.86	14.98	25.48	14.28	32.62	20.45
Trial 2	45.24	18.45	54.29	20.55	43.81	21.71	48.81	22.25
Trial 3	63.57	24.18	70.24	19.42	54.29	21.52	62.38	24.37

Values are the mean study time (in seconds) of words of each type on each trial for participants in the self-paced conditions (those in the computer-paced conditions studied each item for 5 s each) and the mean percentage of words of each type that participants correctly recalled on each trial for all conditions.

We also considered whether study time differed for the computer-paced and self-paced conditions using one-sample *t*-tests. Compared to a fixed study time of five seconds per item on all trials for the computer-paced conditions, participants in the self-paced conditions spent an equivalent amount of time studying both animate and inanimate items on the first study trial (both *p*s > 0.7) but spent significantly less time studying both animate and inanimate items on the second and third study trials (all *p*s < 0.001). These values would remain significant after a Bonferroni correction (*α* = 0.008).

#### 2.2.2. Free-recall performance

We scored recall using a strict criterion as either correct or incorrect. We did not score participants’ recall of the buffer words. We calculated the mean percentage of animate and inanimate words that participants correctly recalled on each trial (Table 2; Figure 1). We analyzed recall with a 2 (animacy: animate vs. inanimate words) × 3 (trial: Trial 1 vs. Trial 2 vs. Trial 3) × 3 (word list source: Nairne et al., 2013 vs. Popp and Serra, 2016 vs. Popp and Serra, 2018) × 2 (study method: computer-paced vs. self-paced) mixed ANOVA. Although participants in the self-paced conditions spent less time studying the items on later trials than did those in the computer-paced conditions, overall levels of recall did not differ based on study method, *F*(1,204) = 0.187, *MSE* = 2078.589, *p* = 0.666, *η_p_^2^* < 0.01. Recall also did not differ by list, *F*(2,204) = 0.121, *MSE* = 2078.589, *p* = 0.886, *η_p_^2^* < 0.01. Participants recalled more animate than inanimate items, *F*(1,204) = 119.469, *MSE* = 235.188, *p* < 0.001, *η_p_^2^* = 0.37. Animacy interacted with list, *F*(2,204) = 6.150, *MSE* = 235.188, *p* = 0.003, *η_p_^2^* = 0.06. Follow-up comparisons indicated that participants recalled more animate than inanimate items for all three lists (all *p*s < 0.001); the effect size was largest for the Nairne et al. (2013) list (*Cohen’s d* = 1.08) and smaller in comparison for the Popp and Serra (2016) list (*Cohen’s d* = 0.65) and the Popp and Serra (2018) list (*Cohen’s d* = 0.54). Polynomial contrasts indicated that recall increased both linearly, *F*(1,204) = 726.191, *MSE* = 241.009, *p* < 0.001, *η_p_^2^* = 0.78, and quadratically, *F*(1,204) = 25.674, *MSE* = 102.484, *p* < 0.001, *η_p_^2^* = 0.11, across the study trials. Unexpectedly, trial interacted with both study method, *F*(2,408) = 11.877, *MSE* = 171.747, *p* < 0.001, *η_p_^2^* = 0.06, and list, *F*(4,408) = 4.521, *MSE* = 171.747, *p* = 0.001, *η_p_^2^* = 0.04; both interactions suggest that gains in memory across trials varied somewhat by the study method and the materials studied. No other interactions were significant.

### 2.3. Discussion

As in past studies, the animacy advantage in free-recall performance occurred for participants in the computer-paced conditions. More important, it also occurred for those in the self-paced conditions, who allocated study time equally to animate and inanimate items. Participants in the self-paced conditions studied the items for less time overall than did those in the computer-paced conditions (and reduced their study time across the trials), but they achieved a comparable level of overall recall compared to participants in the computer-paced conditions. Although past research indicates that some extrinsic or processing differences between animate and inanimate items likely contribute to the animacy advantage (Meinhardt et al., 2020; Rawlinson and Kelley, 2021; Shull et al., n.d.), the present results are consistent with the prior conclusion that under typical settings participants do not seem to be producing this effect purposely, such as by intentionally devoting greater processing effort or depth of processing to animate over inanimate items (*cf.* DeYoung and Serra, 2021; Serra, 2021; Shull et al., n.d.). The self-paced study conditions in the present experiment presented an obvious opportunity for participants to devote greater study time to animate over inanimate items if they chose to do so, but that did not occur. There might still, however, be some conditions under which participants allocate study time differently to animate and inanimate items; we explore this possibility further in Experiment 2.

Direct comparison of the occurrence of the animacy advantage in free-recall performance for the three different word lists indicates that the effect was larger for the Nairne et al. (2013) list than for the other two lists (Popp and Serra, 2016, 2018). As the researchers who created those lists balanced the animate and inanimate items on different factors, it is possible that all three lists contain embedded confounding variables that could moderate the size of the animacy advantage in recall. This does not mean that the entirety of the animacy advantage stems from imbedded confounds between animate and inanimate items, but it does indicate that differences in intrinsic properties between these items can contribute to the effect, especially if left unchecked (*cf.* Popp and Serra, 2018).

## 3. Experiment 2

Participants in the self-paced conditions in Experiment 1 did not devote study time differently to animate versus inanimate items, so the occurrence of the animacy advantage in those experiments cannot be explained by differential study time (although other extrinsic or processing-based mechanisms could of course still have contributed). We found this outcome to be somewhat surprising, given some prior studies have found that task instructions could lead participants to alter the occurrence of the animacy advantage, *even under computer-paced study conditions*. For example, when researchers told participants to expect either an animate or inanimate advantage to occur, participants shifted their encoding to compensate for that expected outcome (DeYoung and Serra, 2021). When researchers told participants to purposely focus on encoding either animate or inanimate items, participants produced an animate or inanimate advantage in free-recall performance, respectively (Shull et al., n.d.).

It is possible that the participants in Experiment 1 did not devote study time differently to animate and inanimate items because their metacognitive beliefs about this effect were not activated prior to (or during) their study of the materials (*cf.* Dunlosky and Tauber, 2014; Tauber et al., 2019). In the present Experiment 2, we attempted to activate their pre-existing metacognitive beliefs about the effect prior to interacting with the materials using a simple metacognitive-beliefs question (a more subtle and perhaps more naturalistic approach than telling participants to expect or to purposely produce a given outcome). Although activating these beliefs prior to encoding could alter the occurrence of the animacy advantage under computer-paced conditions (*cf.* DeYoung and Serra, 2021; Shull et al., n.d.), there is an even greater opportunity for these beliefs to affect study time and the subsequent occurrence of the animacy advantage in the self-paced conditions. Knowing participants’ metacognitive beliefs about the effects of animacy on memory can allow for a more nuanced consideration of the occurrence of the animacy advantage under either study method.

### 3.1. Materials and method

#### 3.1.1. Participants

The participants were 210 undergraduate college students from the psychology participant pool at Texas Tech University. They participated for class credit. None had participated in Experiment 1.

Using the same power analysis as in Experiment 1, we again used 35 participants per group, and the same considerations of power would apply. In this experiment, however, we also considered whether participants’ beliefs might interact with animacy. Assuming an even distribution of beliefs in the sample, we would be sufficiently powered to detect an interaction of animacy and beliefs with a *Cohen’s d* = 0.23 or higher.

#### 3.1.2. Materials

The study materials were the same as in Experiment 1.

#### 3.1.3. Design

The primary design was the same as in Experiment 1: a 2 (animacy: animate vs. inanimate words) × 3 (trial: Trial 1 vs. Trial 2 vs. Trial 3) × 3 (word list source: Nairne et al., 2013 vs. Popp and Serra, 2016 vs. Popp and Serra, 2018) × 2 (study method: computer-paced vs. self-paced) mixed design. The addition of the metacognitive beliefs question, however, allowed us to also examine participants’ beliefs about the effect of animacy on free-recall performance as a group variable.

#### 3.1.4. Procedure

The procedure for Experiment 2 was the same as for Experiment 1 except for the addition of a metacognitive beliefs question at the start of the task. Specifically, after reading the same set of instructions as in Experiment 1, participants in Experiment 2 read the question, “In this experiment, you will be studying a list of twenty-four words for a memory test. Half of the words will represent living (animate) concepts and half will represent non-living (inanimate) concepts. In this experiment, which statement below do you believe will be MOST ACCURATE?.” They responded by picking one of the following options: “I think my memory will be better for living things than non-living things.,” “I think my memory will be equal for living things than non-living things.,” or “I think my memory will be better for non-living things than living things…” After answering this question, participants began the first study trial, and the rest of the procedure proceeded as in Experiment 1. Unlike in Experiment 1, participants in Experiment 2 were therefore made explicitly aware that half of the items would be animate, and half would be inanimate.

### 3.2. Results

The data for Experiment 2 is available at https://osf.io/6kndh/.

#### 3.2.1. Beliefs question

We calculated the number and percentage of participants in the two study method conditions that endorsed each belief prior to beginning the task. In the computer-paced conditions, 41 participants (39.0%) endorsed the belief that animate items would be more memorable than inanimate items, 53 participants (50.5%) endorsed the belief that animate items would be equally as memorable as inanimate items, and 11 participants (10.5%) endorsed the belief that inanimate items would be more memorable than animate items. In the self-paced conditions, 50 participants (47.6%) endorsed the belief that animate items would be more memorable than inanimate items, 45 participants (42.9%) endorsed the belief that animate items would be equally as memorable as inanimate items, and 10 participants (9.5%) endorsed the belief that inanimate items would be more memorable than animate items. The proportions did not differ by study method, *Χ^2^* (2, *N* = 210) = 1.591, *p* = 0.451.

#### 3.2.2. Self-paced study time

We analyzed study time (Table 3; Figure 1) with a 2 (animacy: animate vs. inanimate words) × 3 (trial: Trial 1 vs. Trial 2 vs. Trial 3) × 3 (word list source: Nairne et al., 2013 vs. Popp and Serra, 2016 vs. Popp and Serra, 2018) mixed ANOVA. Self-paced study time did not differ by animacy, *F*(1,102) = 1.119, *MSE* = 1.828, *p* = 0.293, *η_p_^2^* = 0.01, or by list, *F*(2,102) = 0.123, *MSE* = 52.299, *p* = 0.885, *η_p_^2^* < 0.01. Polynomial contrasts indicated that self-paced study time decreased both linearly, *F*(1,102) = 51.101, *MSE* = 38.491, *p* < 0.001, *η_p_^2^* = 0.33, and quadratically, *F*(1,102) = 13.000, *MSE* = 12.908, *p* < 0.001, *η_p_^2^* = 0.11, across the study trials. None of the interactions were significant.

**Table 3:** Mean study time and mean free-recall performance in experiment 2.

List and trial	Computer-paced conditions				Self-paced conditions			
	Inanimate		Animate		Inanimate		Animate		M	SD	M	SD	M	SD	M	SD
Study time (in seconds)
Nairne et al. (2013) List								
Trial 1	5	–	5	–	6.42	7.11	6.23	6.24
Trial 2	5	–	5	–	2.85	3.36	2.61	3.35
Trial 3	5	–	5	–	1.81	1.91	1.47	1.38
Popp and Serra (2016) List								
Trial 1	5	–	5	–	5.27	5.20	4.87	4.50
Trial 2	5	–	5	–	2.82	2.45	2.88	3.88
Trial 3	5	–	5	–	1.94	1.76	1.77	1.53
Popp and Serra (2018) List								
Trial 1	5	–	5	–	6.21	8.33	6.34	7.52
Trial 2	5	–	5	–	2.30	1.94	2.34	2.37
Trial 3	5	–	5	–	1.15	0.68	1.24	0.79
Free-recall performance (% correct)
Nairne et al. (2013) List								
Trial 1	26.19	17.75	45.95	18.34	29.29	24.20	42.62	26.18
Trial 2	46.19	18.89	60.95	18.05	42.38	26.69	56.67	23.12
Trial 3	62.38	21.90	70.71	19.11	55.24	25.57	64.76	21.40
Popp and Serra (2016) List								
Trial 1	32.38	12.58	33.57	14.64	29.05	20.05	36.90	21.03
Trial 2	49.52	20.00	54.29	21.80	47.62	19.23	53.33	20.83
Trial 3	58.81	23.04	65.95	22.08	59.76	16.36	65.95	21.52
Popp and Serra (2018) List								
Trial 1	22.38	13.37	35.00	12.59	31.90	21.81	38.81	25.08
Trial 2	47.38	18.94	53.10	17.75	46.90	21.21	59.76	18.13
Trial 3	56.19	21.80	66.43	20.16	57.62	22.81	70.71	18.46

Values are the mean study time (in seconds) of words of each type on each trial for participants in the self-paced conditions (those in the computer-paced conditions studied each item for 5 s each) and the mean percentage of words of each type that participants correctly recalled on each trial for all conditions.

Compared to a fixed study time of five seconds per item for the computer-paced conditions, participants in the self-paced conditions spent an equivalent amount of time studying both animate and inanimate items on the first study trial (both *p*s > 0.2) but spent significantly less time studying both animate and inanimate items on the second and third study trials (all *p*s < 0.001). These values would remain significant after a Bonferroni correction (*α* = 0.008).

We also repeated the ANOVA above, adding in participants’ beliefs about the effect of animacy on memory (animate > inanimate vs. animate = inanimate vs. animate < inanimate) as a group variable (Figure 1). There was no difference in study time by belief, *F*(2,96) = 0.805, *MSE* = 51.615, *p* = 0.450, *η_p_^2^* = 0.02, nor did belief interact with animacy, *F*(2,96) = 1.476, *MSE* = 1.790, *p* = 0.234, *η_p_^2^* = 0.03. No other effects or interactions were significant either. That said, the main effect of animacy on study time approached significance after accounting for beliefs, *F*(1,96) = 3.146, *MSE* = 1.790, *p* = 0.079, *η_p_^2^* = 0.03, with average study time being slightly higher for inanimate than animate items.

#### 3.2.3. Free-recall performance

We analyzed recall (Table 3; Figure 1) with a 2 (animacy: animate vs. inanimate words) × 3 (trial: Trial 1 vs. Trial 2 vs. Trial 3) × 3 (word list source: Nairne et al., 2013 vs. Popp and Serra, 2016 vs. Popp and Serra, 2018) × 2 (study method: computer-paced vs. self-paced) mixed ANOVA. Although participants in the self-paced conditions spent less time studying the items on later trials than did those in the computer-paced conditions, overall levels of recall did not differ based on study method, *F*(1,204) = 0.002, *MSE* = 1655.462, *p* = 0.963, *η_p_^2^* < 0.01. Recall also did not differ by list, *F*(2,204) = 0.164, *MSE* = 1655.462, *p* = 0.849, *η_p_^2^* < 0.01. Participants recalled more animate than inanimate items, *F*(1,204) = 114.145, *MSE* = 258.722, *p* < 0.001, *η_p_^2^* = 0.36. Animacy interacted with list, *F*(2,204) = 6.358, *MSE* = 258.722, *p* = 0.002, *η_p_^2^* = 0.06. Follow-up comparisons indicated that participants recalled more animate than inanimate items for all three lists (all *p*s < 0.001); the effect size was again largest for the Nairne et al. (2013) list (*Cohen’s d* = 0.98), slightly smaller for the Popp and Serra (2018) list (*Cohen’s d* = 0.84), and smallest for the Popp and Serra (2016) list (*Cohen’s d* = 0.41). Polynomial contrasts indicated that recall increased both linearly, *F*(1,204) = 779.483, *MSE* = 126.073, *p* < 0.001, *η_p_^2^* = 0.79, and quadratically, *F*(1,204) = 19.693, *MSE* = 148.718, *p* < 0.001, *η_p_^2^* = 0.09, across the study trials. Unexpectedly, the triple interaction between animacy, trial, and list was significant, *F*(4,408) = 2.722, *MSE* = 111.149, *p* = 0.029, *η_p_^2^* = 0.03. No other interactions were significant, although the quadruple interaction between animacy, trial, list, and study method approached significance, *F*(4,408) = 2.357, *MSE* = 111.149, *p* = 0.053, *η_p_^2^* = 0.02.

We also repeated the ANOVA above, adding in participants’ beliefs about the effect of animacy on memory (animate > inanimate vs. animate = inanimate vs. animate < inanimate) as a group variable (Figure 1). A difference in the level of recall by beliefs approached significance, *F*(2,192) = 2.486, *MSE* = 1606.375, *p* = 0.086, *η_p_^2^* = 0.03, as overall recall was somewhat lower for those participants who endorsed the belief that animate items are more memorable than inanimate items. Follow up analyses indicated that the trend stemmed from the recall of inanimate items being lower for those who believed that animate items are more memorable than inanimate items than for those who believe that inanimate items are more memorable than animate items (*p* = 0.031) and those who endorsed no difference (*p* = 0.092). Put differently, participants who believed that animate items are more memorable than inanimate items had lower recall of *inanimate* items compared to those who believed otherwise. The interaction between animacy and beliefs approached significance, *F*(2,192) = 2.866, *MSE* = 256.867, *p* = 0.059, *η_p_^2^* = 0.03, as did the interaction between animacy, study method, and beliefs, *F*(2,192) = 2.523, *MSE* = 256.867, *p* = 0.083, *η_p_^2^* = 0.03. As well, the interaction between animacy, trial, study method, list and beliefs was significant, *F*(8,384) = 2.719, *MSE* = 110.548, *p* = 0.006, *η_p_^2^* = 0.05. No other interactions with beliefs approached significance, and the inclusion of beliefs did not greatly alter other outcomes from the prior ANOVA.

Given there seemed to be a difference in whether the effect of animacy was altered by beliefs based on how participants studied the items, we performed two separate 2 (animacy) × 3 (trial) × 3 (word list source) × 3 (beliefs) mixed ANOVAs, split by study method (Figure 1). In this case, beliefs affected participants’ overall level of recall under self-paced conditions, *F*(2,96) = 3.790, *MSE* = 1953.195, *p* = 0.026, *η_p_^2^* = 0.07, but not under computer-paced conditions, *F*(2,96) = 0.089, *MSE* = 1259.554, *p* = 0.915, *η_p_^2^* < 0.01. Whereas there were no significant differences in level of recall by belief within the computer-paced conditions (all *p*s > 0.76), the pattern within the self-paced conditions mirrored the pattern obtained in the larger ANOVA: the effect stemmed largely from the recall of inanimate items being higher for those who believed that inanimate items are more memorable than animate items than for those who believed that animate items are more memorable than inanimate items (*p* = 0.002), although recall was also higher for those who believed that inanimate items are more memorable than animate items compared to those who endorsed no difference (*p* = 0.080). As well, inanimate recall was somewhat higher for those who endorsed no difference compared to those who believed that animate items are more memorable than inanimate items (*p* = 0.099). Within the computer-paced conditions, animacy interacted with list, *F*(2,96) = 3.568, *MSE* = 274.368, *p* = 0.032, *η_p_^2^* = 0.07, but not with beliefs, *F*(2,96) = 0.760, *MSE* = 274.368, *p* = 0.470, *η_p_^2^* = 0.02. In contrast, within the self-paced conditions, animacy interacted with beliefs, *F*(2,96) = 4.488, *MSE* = 239.366, *p* = 0.014, *η_p_^2^* = 0.09, but not with list, *F*(2,96) = 1.184, *MSE* = 239.366, *p* = 0.310, *η_p_^2^* = 0.02. More specifically, in the computer-paced conditions, the effect size for animacy was largest for the Nairne et al. (2013) list (*Cohen’s d* = 1.06), somewhat smaller for the Popp and Serra (2018) list (*Cohen’s d* = 0.77), and smallest for the Popp and Serra (2016) list (*Cohen’s d* = 0.31). In the self-paced conditions, the effect size for animacy was largest for participants who endorsed no difference (*Cohen’s d* = 0.93), slightly smaller for those who believed that animate items are more memorable than inanimate items (*Cohen’s d* = 0.84), and smallest (and trending in the other direction) for participants who believed that inanimate items are more memorable than animate items (*Cohen’s d* = −0.14).

### 3.3. Discussion

Overall, the results of Experiment 2 were consistent with those of Experiment 1. We obtained the animacy advantage in free-recall performance, again regardless of study method (computer-paced versus self-paced study). Although participants in the self-paced conditions devoted less total study time to items compared to those in the computer-paced conditions, overall recall was again the same regardless of study method, and participants in the self-paced conditions again did not devote time differently to animate versus inanimate items.

In terms of participants’ metacognitive beliefs, it does not seem that having participants self-report their metacognitive beliefs prior to beginning the task altered the occurrence of the animacy advantage in the computer-paced conditions, replicating outcomes from similar situations in some of the experiments reported by DeYoung and Serra (2021). In contrast, although participants in the self-paced conditions demonstrated about the same level of recall of animate items regardless of their beliefs, their recall of the inanimate items increased from those who endorsed the belief that animate items would be more memorable than inanimate items to those who endorsed the belief that animate items would be equally as memorable as inanimate items, and again to those who endorsed the belief that inanimate items would be more memorable than animate items. This pattern occurred without major differences in study time by group or animacy, which contradicts our prediction that participants might allocate their study time to the items differently based on their metacognitive beliefs. Nevertheless, these outcomes indirectly support extrinsic, processing-based accounts of the animacy advantage in free-recall performance, as participants with different beliefs about the effect of animacy on memory presumably processed the inanimate items differently in order to produce the different levels of recall of these items. As in several prior studies (e.g., Bonin et al., 2015; Leding, 2018; DeYoung and Serra, 2021; Shull et al., n.d.), the effects of this processing were most noticeable on inanimate items, again suggesting that animate items normally trigger greater processing than inanimate items, regardless of people’s beliefs, but that people can increase their processing of inanimate items to remember more of them.

## 4. General discussion

### 4.1. Effects of study method

In both Experiments 1 and 2, we examined the animacy advantage in free-recall performance under computer-paced versus self-paced study conditions and using three different sets of animate and inanimate stimuli. We also considered whether participants’ metacognitive beliefs (i.e., expectations) about the task—which we measured at the onset of the procedure in Experiment 2—altered the effects of animacy on study time or recall.

In both experiments, we obtained the animacy advantage in free-recall performance, regardless of whether participants studied the materials under computer-paced or self-paced conditions. Even though participants in the self-paced conditions spent less total time studying the items than did participants in the computer-paced conditions, the overall levels of recall and the occurrence of the animacy advantage were equivalent for the two study methods. Importantly, participants tended to devote equivalent study time to animate and inanimate items in the self-paced conditions, so the occurrence of the animacy advantage in those conditions cannot be attributed to a difference in study-time allocation (Surprisingly, total study time was lower for the self-paced conditions than for the computer-paced conditions, yet the overall level of recall was the same). The results indirectly support at least two prior suppositions about the animacy advantage: (1) under most conditions, participants do not seem to purposely produce the animacy advantage by devoting greater processing to animate over inanimate items (*cf.* DeYoung and Serra, 2021; Serra, 2021), and (2) animate items seem to trigger greater richness of encoding than do inanimate items (*cf.* Meinhardt et al., 2020; Rawlinson and Kelley, 2021; Bonin et al., 2022), likely outside of participants’ awareness or control (DeYoung and Serra, 2021). Regarding this latter point, however, the present studies do not elucidate the form of this additional (or different) form of encoding or processing. Future research should continue to work to identify factors related to animacy that alter processing, or greater forms of processing such as spreading activation, that might contribute to the effect (e.g., Meinhardt et al., 2020; Rawlinson and Kelley, 2021).

### 4.2. Metacognitive beliefs

Much as in DeYoung and Serra (2021), in the present Experiment 2, participants’ pre-existing beliefs about the animacy advantage did not relate to the occurrence of the effect when participants encoded the items under computer-paced study conditions, as well as for most participants in the self-paced conditions. That said, participants in the self-paced study conditions who believed that inanimate items would be more memorable than animate items studied all items for longer than did other participants, devoted equal study time to animate and inanimate items, and ultimately showed *equivalent* recall for animate and inanimate items. These results are in line with those of other studies that implicitly or explicitly encouraged participants to devote extra—and equivalent—processing effort to animate and inanimate items, resulting in a reduced or eliminated animacy advantage. For example, Bonin et al. (2015, Study 4) instructed their participants to produce interactive mental imagery during study, which reduced the size of the animacy advantage (primarily by increasing the recall of inanimate items compared to no interactive imagery instructions). Shull et al. (n.d.) crossed high and low point values (points earned for correctly recalling each item) with animate and inanimate items; an animacy advantage still occurred for low-value items, but the recall of high-value items was higher and showed no difference by animacy. Such outcomes suggest that—by default—animate items might trigger more processing than inanimate items, but conditions that encourage participants to devote greater or equivalent processing to inanimate items can reduce, eliminate, or even reverse (Shull et al., n.d.) the occurrence of the animacy advantage in free-recall. It is not immediately clear why participants who believed inanimate items were more memorable than animate items obtained equivalent recall for animate and inanimate items after studying them for the same amount of time, whereas most other participants studied animate and inanimate items for the same amount of time but still demonstrated an animacy advantage; presumably, this subset of participants processed the inanimate items in a different way than did other participants which led to enhanced recall for the inanimate items.

In the present experiments, we utilized participants’ pre-existing beliefs about the effects of animacy on memory rather than trying to manipulate their beliefs. Although considering pre-existing beliefs prevents us from making causal conclusions and produces groups of unequal size, we know from prior research that trying to manipulate these beliefs is ineffective and produces unexpected effects. More specifically, DeYoung and Serra (2021, Experiment 2) attempted to manipulate participants’ beliefs about the effects of animacy on free-recall performance by telling participants at the onset of the task to either expect to recall more animate than inanimate items, to recall more inanimate than animate items, to recall an approximately even number of animate and inanimate items, or they provided no expectation. In addition, their participants made metacognitive memory judgments for every item they studied. The provided beliefs did not affect participants’ judgments of their memory (i.e., all groups judged animate items as more memorable than inanimate items regardless of the beliefs provided), which suggests that the provided information likely had little or no effect on their beliefs about animacy. Unexpectedly, however, the beliefs altered the effect of animacy on free-recall performance. The typical animacy advantage occurred for the group not given any expectation and for the group told to expect to recall more *inanimate* than animate items. Recall, however, did *not* differ by animacy for the group told to expect to recall more animate than inanimate items or the group told to expect no difference. Even though encoding was computer-paced in that experiment, participants were apparently able to alter their encoding effort or strategy to compensate for the provided outcomes to some extent (*cf.* Shull et al., n.d.).

### 4.3. List effects

The three lists we used in the present experiments all consistently produced an animacy advantage in free-recall performance, even when that effect was moderated by other factors. The size of that advantage, however, consistently differed by list (Tables 2, 3). Combining the experiments (*n* = 420), the Nairne et al. (2013) list produced the largest animacy advantage (*Cohen’s d* = 1.03), the Popp and Serra (2018) list produced a somewhat smaller animacy advantage (*Cohen’s d* = 0.68), and the Popp and Serra (2016) list produced the smallest effect (*Cohen’s d* = 0.52). As previously shown in the context of paired associates learning (Serra and DeYoung, 2023), the selection of animate and inanimate stimuli can moderate the effects of animacy on memory. We do believe that animacy somehow aids the recall of single words in the free-recall paradigm, but researchers must understand that other factors besides animacy might also be contributing to the results obtained in any experiment. More positively, we hope that future research can more deeply examine how specific factors moderate the effects of animacy on memory, not just as potential confounding factors, but perhaps as hints to identify the mechanism(s) responsible for this effect. For example, as previously suggested by Popp and Serra (2018), the rather large animacy advantage produced by the Nairne et al. (2013) list might be partially attributable to the fact that those animate stimuli are more mentally arousing than are those inanimate stimuli, even though those Popp and Serra (2018) found an animacy advantage for items matched on arousal. As well, consider that the Popp and Serra (2016) list produced the smallest effect size in the present experiments, even though those animate and inanimate stimuli were matched on fewer factors than the Nairne et al. (2013) and Popp and Serra (2018) lists. Rather than producing a set of animate items that were favored for recall by other factors in addition to animacy, it is possible that the Popp and Serra (2016) list contains a confounding factor(s) that favors the recall of the *inanimate items,* reducing the obtained size of the animate advantage for this list. There are also some apparent differences in the attributes of the words across the three lists that could have contributed to the differing size of the animacy advantage across the lists (Table 1). For example, estimated age of acquisition is noticeably lower for words in the Nairne et al. (2013) list than in the other two lists. Although the animate and inanimate words were balanced on factors *within* each list, it is possible that animacy interacts with some of these factors in yet-unidentified ways. We recommend that researchers continue to examine the contribution of various factors besides animacy to recall using more continuous analyses such as regression or modeling (*cf.* Nairne et al., 2013; see also Gelin et al., 2017; VanArsdall and Blunt, 2022), and to consider whether those factors are independent from animacy or not.

### 4.4. Future directions

Going forward, we recommend that researchers consider two major classes of explanation for the animacy advantage: one class that focuses on *intrinsic* or *item-level* differences in memory-relevant factors that might exist between animate and inanimate words and could contribute to the effect, and one class that focuses on *extrinsic* or *processing* differences between animate and inanimate words (Rawlinson and Kelley, 2021, referred to such accounts as *controlled processing*) that could contribute to the effect. For example, many of the accounts for the animacy advantage which have already been discredited would fall into the intrinsic category: the effect does not seem to occur because animate and inanimate words differ on threat (Leding, 2019, 2020), arousal (Meinhardt et al., 2018; Popp and Serra, 2018; Leding, 2019), or categorizability (Gelin et al., 2017; VanArsdall et al., 2017; Serra, 2021). Some of the currently more viable accounts would fall into the extrinsic category: the effect might occur because animate items activate more related information (Meinhardt et al., 2020; Bonin et al., 2022) or have more semantic features (Rawlinson and Kelley, 2021). Admittedly, some factors, such as attentional capture, do not fall neatly into either an intrinsic or extrinsic category. As well, intrinsic and extrinsic mechanisms need not be exclusive and could even work together to produce the effect (*cf.* Meinhardt et al., 2020). Going forward, however, we think there can be value in separating tentative accounts of the animacy advantage into these two categories when possible. As a growing body of data discredits intrinsic or item-level accounts of the effect but supports extrinsic or processing-based accounts, it might be more efficient for researchers to focus on testing hypotheses for the effect that favor extrinsic differences between animate and inanimate words rather than intrinsic differences.

As well, researchers could examine whether the animacy advantage in memory differs across different levels and even “types” of animacy, rather than treating animacy as a living-vs.-nonliving binary as we did in the present report (and most other researchers have done as well). For example, VanArsdall and Blunt (2022) identified several subfactors related to people’s concepts of animacy, such as thought, movement, reproduction, goal setting, and similarity to humans. These factors might relate to the memorability of a given concept to different degrees, or even interact.

## Data availability statement

The datasets presented in this study can be found in online repositories. The names of the repository/repositories and accession number(s) can be found at: https://osf.io/6kndh/.

## Ethics statement

The studies involving human participants were reviewed and approved by Texas Tech University IRB and HRPP. The patients/participants provided their written informed consent to participate in this study.

## Author contributions

MS and CD devised the study, collected and analyzed the data, and wrote the manuscript. All authors contributed to the article and approved the submitted version.

## Conflict of interest

The authors declare that the research was conducted in the absence of any commercial or financial relationships that could be construed as a potential conflict of interest.

## Publisher’s note

All claims expressed in this article are solely those of the authors and do not necessarily represent those of their affiliated organizations, or those of the publisher, the editors and the reviewers. Any product that may be evaluated in this article, or claim that may be made by its manufacturer, is not guaranteed or endorsed by the publisher.
